# *Lacticaseibacillus rhamnosus* AC1 Aggravates Bone Loss in a Male Rat Model of Deoxycorticosterone Acetate (DOCA)-Salt-Induced Osteoporosis

**DOI:** 10.3390/nu17203198

**Published:** 2025-10-11

**Authors:** Xiaoqing Kuang, Haicui Wu, Tim Fat Shum, Chunyi Wen, Jiachi Chiou

**Affiliations:** 1 Department of Food Science and Nutrition, Faculty of Science, The Hong Kong Polytechnic University, Hung Hom, Hong Kong SAR, China; xiaoqing7.kuang@connect.polyu.hk (X.K.); haicui123.wu@polyu.edu.hk (H.W.); tf-perry.shum@polyu.edu.hk (T.F.S.); 2 Department of Biomedical Engineering, Faculty of Engineering, The Hong Kong Polytechnic University, Hung Hom, Hong Kong SAR, China; 3 Research Institute of Smart Ageing, The Hong Kong Polytechnic University, Hung Hom, Hong Kong SAR, China; 4 Research Institute for Future Food, The Hong Kong Polytechnic University, Hung Hom, Hong Kong SAR, China

**Keywords:** osteoporosis, probiotics, bone remodeling, gut microbiota

## Abstract

**Background/Objectives**: Osteoporosis is a prevalent and debilitating skeletal disease characterized by a progressive loss of bone mass and deterioration of bone microarchitecture. Probiotics have emerged as a potential therapeutic tool for treating osteoporosis through modulation of the gut microbiota. In this study, we aimed to examine the effects of live *Lacticaseibacillus rhamnosus* AC1 (LR-AC1), isolated from a fecal sample from a newborn in Hong Kong, on deoxycorticosterone acetate (DOCA)-induced bone loss in a rat model. **Methods**: Bone mass and microarchitecture were assessed using micro-computed tomography (micro-CT). Immunostaining for CD31^+^ and osterix, markers of endothelial cells and osteoblast precursors, respectively, was performed. Gut microbiota composition was analyzed via 16S rRNA sequencing. The effects of an LR-AC1 cell-free conditioned supernatant (CCS) on osteoclastogenesis, angiogenesis, and migration of bone marrow mesenchymal stem cells (BMSCs) were evaluated in vitro using RT-qPCR and wound healing assays. **Results**: LR-AC1 administration did not induce adverse effects in healthy rats; however, it exacerbated bone loss in rats with DOCA-salt-induced osteoporosis. Correspondingly, the number of CD31-positive endothelial cells and osterix-positive osteoprogenitors decreased with bone loss. In vitro, LR-AC1 CCS promoted osteoclastogenesis and angiogenesis, while in the presence of DOCA, LR-AC1 CCS inhibited BMSC migration. Gut microbiota analysis revealed that the relative abundances of the genera *g_RF39* and *g_Clostridia_UCG-014* correlated with the severity of bone loss. **Conclusions**: While several studies suggest that probiotics can prevent and treat osteoporosis, our findings indicate that in a male rat model of DOCA-salt-induced osteoporosis, live LR-AC1 aggravated bone loss. This effect is associated with alterations in gut microbiota and disruption of the coupling process in bone remodeling.

## 1. Introduction

Osteoporosis is a prevalent, debilitating, and often silent skeletal disease characterized by a loss of bone mass, compromised bone strength, and an increased risk of fracture [1]. It results from an imbalance between osteoclast-mediated bone resorption and osteoblast-mediated bone formation. In addition to conventional pharmaceutical treatment such as bisphosphonates and teriparatide, which are associated with significant side effects, nutrient supplements have gained increasing public attention as potential therapies for osteoporosis.

The gut microbiota is in proximity to various cell types and influences multiple physiological systems, including the intestinal nervous and immune systems, enteroendocrine function, and enterocyte permeability. It also contributes to the production of vitamins and secondary bile salts, competes with pathogenic microorganisms to prevent their invasion, and stimulates immune responses [2,3]. The gut microbiota comprises diverse microorganisms, including probiotics, which are live and non-pathogenic microorganisms that, when administered in adequate amounts, confer health benefits to the host [4].

Mounting evidence suggests that probiotics may have preventive and therapeutic effects on osteoporosis. A randomized, double-blind, placebo-controlled clinical trial involving 50 patients aged 50 to 72 with bone loss demonstrated that supplementation with multiple probiotics positively affected bone health in menopausal women by reducing the rate of bone turnover [5]. A recent study showed that *Lactobacillus reuteri* could ameliorate osteoporosis induced by type 1 diabetes in a mouse model by preventing TNF-α-mediated inhibition of Wnt10b and markers of osteoblast maturation [6]. Additionally, McCabe et al. reported that supplementation with *L. reuteri* ATCC PTA 6475 improved trabecular bone parameters of the femur and lumbar vertebrae in male mice with intestinal inflammation [7]. Collectively, current evidence supports the potential of probiotics to treat secondary osteoporosis associated with postmenopausal estrogen deficiency, type 1 diabetes, and intestinal inflammation. However, the effects of *Lactobacillus* on secondary osteoporosis following long-term steroid treatment remain under investigation [8].

In this study, deoxycorticosterone acetate (DOCA), an adrenocortical hormone and corticosteroid used clinically to treat primary adrenal insufficiency and inflammation, was employed as a model to induce hypertension [9,10]. Notably, steroid use is a recognized causative factor contributing to osteoporosis and has a high incidence among individuals with this condition [8]. Many chronic inflammatory diseases require steroids for treatment, yet long-term steroid use or short-term use of high-dose steroids may result in bone loss. We utilized a strain of *Lacticaseibacillus rhamnosus* isolated from a fecal sample from a healthy infant from Hong Kong, which was previously applied in hypertension treatment, to investigate whether supplementation with this strain can ameliorate DOCA-salt-induced osteoporosis and its associated bone alterations.

## 2. Materials and Methods

### 2.1. Bacterial Culture

LR-AC1 was isolated from the fecal sample of a healthy infant from Hong Kong [11]. LR-AC1 was cultured in De Man, Rogosa and Sharpe (MRS) Broth, and its identity was confirmed by 16S ribosomal RNA (rRNA) sequencing.

### 2.2. Animal Experiment

Sprague Dawley (SD) rats were bred by the Centralized Animal Facilities (CAFs) at The Hong Kong Polytechnic University. To establish a DOCA-salt-induced rat model of osteoporosis, male rats aged 6 weeks and weighing between 180 and 200 g were used. The animal experiment was approved by the Animal Subjects Ethics Committee of The Hong Kong Polytechnic University (ASESC No: 17-18/50-ABCT-R-HMRF) [9,12]. All experimental procedures and licenses were approved and granted by the Hong Kong Department of Health. The rats were housed at the CAFs under controlled conditions at a temperature of 21 ± 2 °C, relative humidity of 55% ± 10%, and a 12 h light/dark cycle. Standard diet and water were provided ad libitum. Prior to the start of the experiment, all animals were acclimated to the experimental environment for 2 weeks. Cage randomization and treatment administration were blinded to reduce bias.

**Figure 1:**
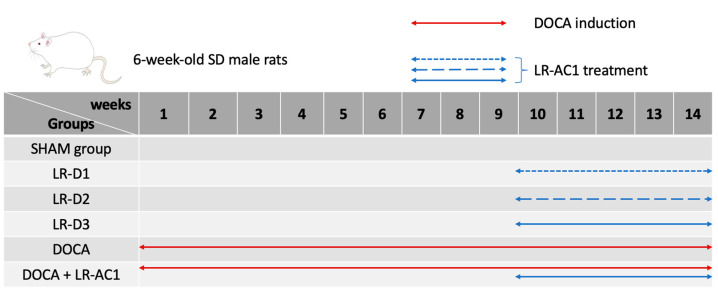
Design of animal experiment. Standard diet and water were provided to the SHAM group (*n* = 11) and the LR-D1 (*n* = 6), LR-D2 (*n* = 5), and LR-D3 (*n* = 6) groups for 14 weeks. Different dosages of live LR-AC1 were administered to the rats by oral gavage starting from week 10, and the treatment lasted for 5 weeks. DOCA treatment was administered to the DOCA group (*n* = 8) and the DOCA + LR-AC1 group (*n* = 8) for 14 weeks. During the last 5 weeks, 10^10^ CFU/kg b.w. of live LR-AC1 was given to the DOCA + LR-AC1 group.

To investigate the effect of LR-AC1 on healthy rats, the SD rats were orally administered an LR-AC1 suspension in 0.85% NaCl daily at dosages of 10^8^, 10^9^, or 10^10^ CFU/kg b.w., according to which they were grouped as LR-D1, LR-D2, or LR-D3, respectively. The dose and duration of LR-AC1 administration followed a previous study in *Lacticaseibacillus rhamnosus* [13,14]. The SHAM group received tap water. The treatment period lasted for 5 weeks (Figure 1).

Hypertension was induced by intraperitoneal injection of DOCA (20 mg/kg b.w., Sigma, D7000, St. Louis, Missouri, USA) twice weekly, in combination with 1.0% NaCl and 0.2% KCl administered in drinking water, while the SHAM group received tap water. The treatment period lasted for 14 weeks.

To investigate the effect of LR-AC1 on the rat model of DOCA-salt-induced osteoporosis, 10^10^ CFU/kg b.w. of LR-AC1 suspension in 0.85% NaCl was administered every day by oral gavage to the rats that had undergone DOCA treatment for 9 weeks. The probiotic treatment lasted for 5 weeks (Figure 1). At the end of the experiment, the rats were anesthetized with CO_2_ for subsequent analyses. Both the left and right legs were stored in 75% ethanol after fixation for 48 h. Fecal samples were collected weekly and immediately following anesthesia and stored at −80 °C.

### 2.3. 16S rRNA Sequencing

Total genomic DNA was extracted from the collected fecal samples using the TIANamp Stool DNA Kit (TIANGEN, Beijing, China). The V3–V4 region of the 16S rRNA gene was amplified using the 16S-338F forward primer and 16S-806R reverse primer [9]. Sequencing of the 16S rRNA gene was performed by Majorbio Bio-pharm Technology Co., Ltd. (Shanghai, China), using the Illumina HiSeq 2000 platform (Illumina, Inc., San Diego, CA, USA). For post-sequencing analysis, primer sequences were trimmed, and raw data were processed using QIIME 2 with high-performance computing resources provided by the Information Technology Services Office, The Hong Kong Polytechnic University. Paired-end sequences with high-quality reads were denoised using the DADA2 plugin, and taxonomy was assigned based on the SILVA database. Alpha diversity indices and beta-diversity weighted UniFrac principal coordinate analysis (PCoA) plots were generated using QIIME 2 pipelines.

### 2.4. High-Resolution Micro-CT Scanning and Analysis

At the end of the treatment, all rats were euthanized, and their legs were harvested for further analyses. Micro-CT scanning was conducted at the Department of Orthopedics and Traumatology, The University of Hong Kong. The right tibias were scanned using an in vivo X-ray microtomograph Skyscan 1076 (SkyScan n.v., Aartselaar, Belgium). Images were acquired with an X-ray tube voltage of 88 kV, a current of 100 µA, and a Al1.0 mm filter. The exposure time was 560 ms, with a rotation step size of 0.600 degrees. The micro-CT pixel size was 9 µm. Image reconstruction was performed using NRecon software.

The primary spongiosa of the right tibia was analyzed by Micro-CT 3D.SUITE software (BRUKER, Billerica, MA, USA), which includes DATAVIEW and CTAN. The images were saved as Recon files and opened with Dataview (version 1.4.4.0), with the position adjusted to ensure they all had the same direction. A single volume of interest, which covered all the parts to be analyzed, was selected. CTAN was used to further analyze the images. In brief, the volume of interest saved from Dataview was applied. The top and bottom of the selection were chosen. For the primary spongiosa, the top of the selection was the first image in which the trabecular bone of the primary spongiosa appeared in full. A total of 200 images were counted, and the 200th image was the bottom of the selection. The region of interest was drawn, and the dataset of the region of interest was saved to create a 3D model. Finally, 3D analysis was performed in Morphometry preview to determine the percent bone volume, trabecular thickness, trabecular number, and trabecular separation, which were saved.

### 2.5. Tissue Processing

The bone samples were fixed in 4% Paraformaldehyde (PFA) solution for 48 h, followed by storage in 75% alcohol. A 10% Ethylene Diamine Tetraacetic Acid (EDTA) solution was used for decalcification, and the solution was changed every four days until the bone samples softened. The bone samples were then placed in a Leica TP1020 tissue processor (Nussloch, Germany) for processing. They were embedded in paraffin using a Leica ARCADIA H (Nussloch, Germany) until they cooled down. The embedded samples were then cut into 5 µm thick sections for staining.

### 2.6. Immunohistochemistry for Osterix and CD31^+^

Immunohistological staining for Osterix (Sp7) and CD31^+^ was performed using the sections of embedded tissue described previously. After deparaffinization and rehydration, the slices were placed in a citrate buffer at 90 °C for 10 min to retrieve the antigen for Osterix staining or placed in a Tris/EDTA buffer at a pH of 9.0 and 90 °C–95 °C for 3 min for CD31^+^ staining. Then, the sections were treated with 3% hydrogen peroxide for 10 min in the dark, followed by blocking with 10% horse serums. The primary antibodies Sp7 (Cat# ab209484, Abcam, Cambridge, UK) (1/1000) and CD31^+^ (Cat# ab182981, Abcam) (1/1000) were co-incubated with the sections at 4 °C overnight, following the manufacturer’s instructions. At the same time, the negative control samples were incubated with PBS. A Vectastain ABC kit and a DAB peroxidase substrate kit (Vector Labs, Newark, CA, USA) were used to stain the targeted antigen for 3,3′-Diaminobenzidine (DAB). The sections were then subjected to hematoxylin staining and dehydration. Finally, DPX was used as a mounting medium to mount the slides. Compared to the negative control, the positive signals appeared brown. ImageJ 1.53j (National Institutes of Health, Bethesda, MD, USA) was used to analyze the DAB staining results. For CD31^+^, the area fraction was calculated, and the osterix result was the ratio of nuclei with positive signals to the total number of nuclei.

### 2.7. Preparation of LR-AC1 Cell-Free Conditioned Supernatant (CCS)

A single colony of LR-AC1 was inoculated in MRS broth at 37 °C for 18 h, diluted to 10%, and subcultured in fresh MRS until the OD reached 0.3. The culture was centrifuged at 4000× *g* rpm for 10 min. The cell pellet was collected, washed twice with sterile phosphate-buffered saline (PBS), resuspended in Dulbecco’s modified Eagle medium (DMEM) with an OD600 of 3.0, and incubated at 37 °C for 3 h on a shaker of 60 rpm. The supernatant was collected and passed through a 0.22 µm filter. The fluid was fractionated to include only the fraction < 10 kDa, using a centrifugal Amicon filter unit (Merck Millipore Ltd., Cork, Ireland). The LR-AC1 CCS was pipetted into 96-well plates in 250 µL aliquots, lyophilized, and stored at −80 °C [15].

### 2.8. Treatment of LR-AC1 CCS on Osteoclasts

RAW264.7 TIB-1^TM^ cells were cultured with DMEM containing 10% fetal bovine serum (FBS) in an incubator with a CO_2_ content of 5% at 37 °C. RAW264.7 cells were passaged using a cell scraper and seeded in a 24-well plate at a density of 2 × 10^4^ cells/well for 24 h before treatment. Receptor activator of nuclear factor kappa-B ligand (RANKL), 30 ng/mL, was used to induce osteoclast differentiation. Final concentrations of 0.05%, 0.1%, 0.5%, 1%, 5%, and 10% LR-AC1 CCS were used to treat the induced cells for 24 h for the detection of osteoclast biomarkers, including Matrix metallopeptidase 9 (MMP-9), Cathepsin K (Ctsk), and Tartrate-resistant acid phosphatase (TRAP), and the angiogenesis biomarker Platelet-derived growth factor A (PDGF-A). The same treatment procedure was used to detect the biomarkers of angiogenesis Platelet-derived growth factor B (PDGF-B), except the treatment time with LR-AC1 CCS was 36 h. The cells were collected for RNA extraction at the end of the experiment. There were three biological and technical replicates each.

### 2.9. Reverse Transcriptase Quantitative Polymerase Chain Reaction (RT-qPCR)

After RNA extraction, cDNA synthesis was performed using PrimeScript RT Master Mix (TaKaRa Cat# RR036A, Shiga, Japan). The mixtures were incubated first at 37 °C for 15 min for reverse transcription, then at 85 °C for 5 sec to inactivate the reverse transcriptase, and then cooled down to 4 °C. The cDNA samples were stored at −20 °C for future use. The qPCR was performed using the QuantStudio™ 7 Flex Real-Time PCR System (Waltham, MA, USA). The primer sequences for the targets, including PDGF-A, PDGF-B, MMP-9, Ctsk, and TRAP, are shown in Table 1.

### 2.10. Wound Healing Assay of BMSCs Co-Cultured with RAW264.7

Transwell was used to perform the wound healing assay. BMSC PCS-500-012^TM^ (6 × 10^4^ cells/well) cells were seeded in a 24-well cell culture plate, while RAW264.7 (1 × 10^4^ cells/well) cells were seeded in the transwell insert for incubation at 37 °C for 24 h. A 1000 µL pipette tip was used to create a gap in the BMSC culture layer. The culture medium was removed, and treatment mediums (Table 2) were added to both the transwell and insert. Pictures were taken before and after treatment under a microscope (OLYMPUS CKX53, Tokyo, Japan). Image J (National Institutes of Health, Bethesda, MD, USA) was used to measure the gap area. There were three biological and technical replicates each.

### 2.11. Statistical Analyses

GraphPad Prism 8 was used for all statistical analyses. All the results are presented as means ± SEM. One-way analysis of variance (ANOVA) with Tukey’s test was used to compare multiple values from different groups. *p* values less than 0.05 were considered significant. Pearson correlation coefficients were used for correlation analysis.

## 3. Results

### 3.1. LR-AC1 Treatment Aggravates DOCA-Salt-Induced Bone Loss in Osteoporotic Rats but Not in Healthy Rats

Three-dimensional model images of the tibial primary spongiosa from the SHAM, live LR-AC1 treatment, DOCA treatment, and DOCA + LR-AC1 treatment groups were obtained using micro-CT. The bone parameters of the trabecular bone, including the ratio of bone volume to total volume (BV/TV), thickness (Tb.Th), number (Tb.N), and separation (Tb.Sp), were analyzed. The BV/TV ratio indicates the density of the bone, while Tb.Sp refers to the distance between the adjacent trabecular bones. All these parameters reflect the extent of osteoporosis and help predict the risk of fracture.

**Figure 2:**
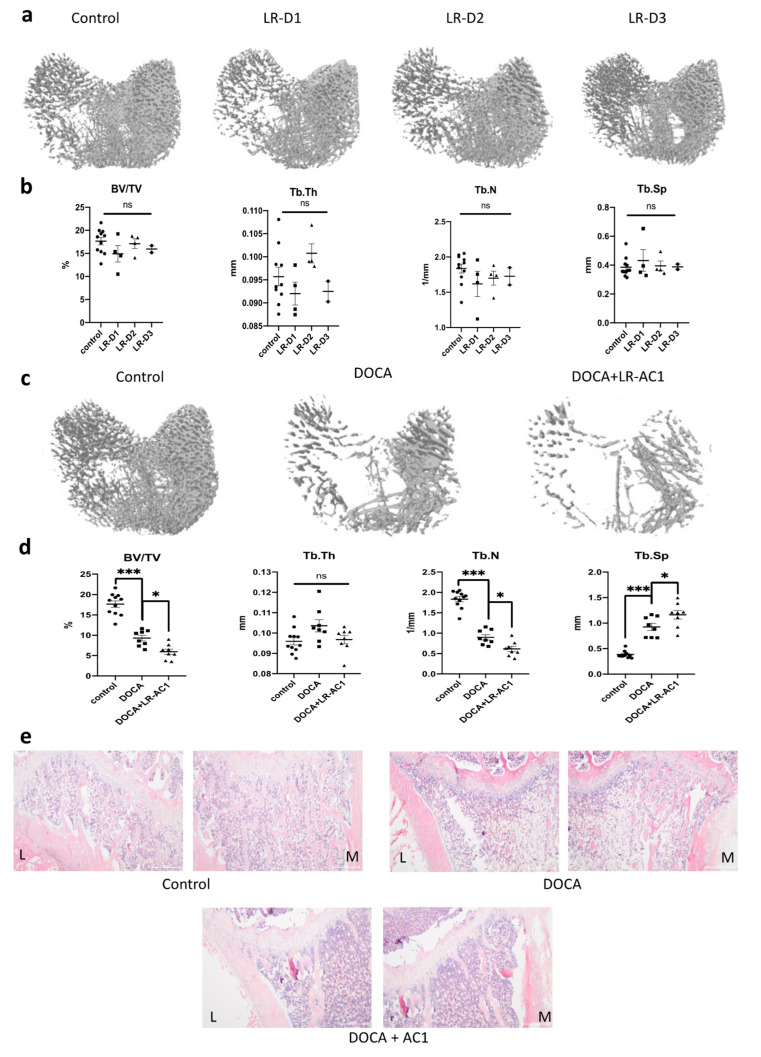
Effect of live LR-AC1 and DOCA on the primary spongiosa of tibia by micro-CT analysis and H&E staining. Micro-CT structures of control group and treatment groups supplemented with 3 dosages of live LR-AC1 (**a**) and their corresponding bone parameters (**b**) Micro-CT structures of SHAM rats and DOCA rats with or without live LR-AC1 supplementation (**c**) and their corresponding bone parameters. (**d**) H&E staining of tibia primary spongiosa of SHAM rats and DOCA rats with or without live LR-AC1 supplementation. Scale bar, 500 μm (**e**) LR-D1, D2, and D3, live LR treatment of 1 mL/kg rat body weight of 10^8^, 10^9^, or 10^10^ CFU/mL LR-AC1, respectively. BV/TV, trabecular bone volume to total volume fraction; Tb.Th, trabecular bone thickness; Tb.N. trabecular bone number; Tb.Sp, trabecular bone separation; L, lateral; M, medial. The values are presented as mean ± SEM. Statistical analysis was performed using one-way ANOVA, with significance levels indicated as follows: *, 0.01 < *p* < 0.05; ***, *p* < 0.001. ns, no significance.

To evaluate the effect of live LR-AC1 on the bone phenotype, three dosages of live LR-AC1, namely 10^8^, 10^9^, or 10^10^ CFU/kg b.w., were administered to healthy SD rats. The 3D model images showed that LR-AC1 treatment did not affect the macro-structure of the primary spongiosa (Figure 2a). Further analysis confirmed that there were no significant differences in bone parameters, including BV/TV, Tb.Th, Tb.N, and Tb.Sp, among the SHAM group and the three groups receiving different dosages of LR-AC1 (Figure 2b). These results indicated that live LR-AC1 had a neutral effect on the bone phenotype of healthy rats.

DOCA was used to induce osteoporosis, and bone loss was observed in the 3D model images and H&E staining (Figure 2c,e). More complete trabecular bones, particularly on the lateral side, were observed in the SHAM group compared to the DOCA treatment group. Both BV/TV and Tb.N showed significant reductions of 47.32% and 51.05% following osteoporosis induction (*p* < 0.0001 and *p* < 0.0001, respectively), while Tb.Sp increased 2.4-fold (*p* < 0.0001). No changes in Tb.Th were observed among the different groups. In summary, these results demonstrate that DOCA successfully induced bone loss. In the rats with DOCA-induced osteoporosis treated with live LR-AC, BV/TV and Tb.N decreased significantly to approximately 35.88% and 31.74% (*p* = 0.0176 and 0.0181, respectively), while Tb.Sp increased 1.26-fold (*p* = 0.0280) (Figure 2d). Consistent with these findings, the 3D model images and histological staining also revealed bone loss in these rats. Fewer trabecular bones were observed in the rats supplemented with LR-AC1 compared to the DOCA group. Collectively, these results indicate that live LR-AC1 treatment had no obvious effect on healthy rats yet aggravated bone loss in rats subjected to DOCA induction.

### 3.2. LR-AC1 Treatment Aggravates DOCA-Salt-Induced Bone Loss, but Healthy Rats Are Unaffected

**Figure 3:**
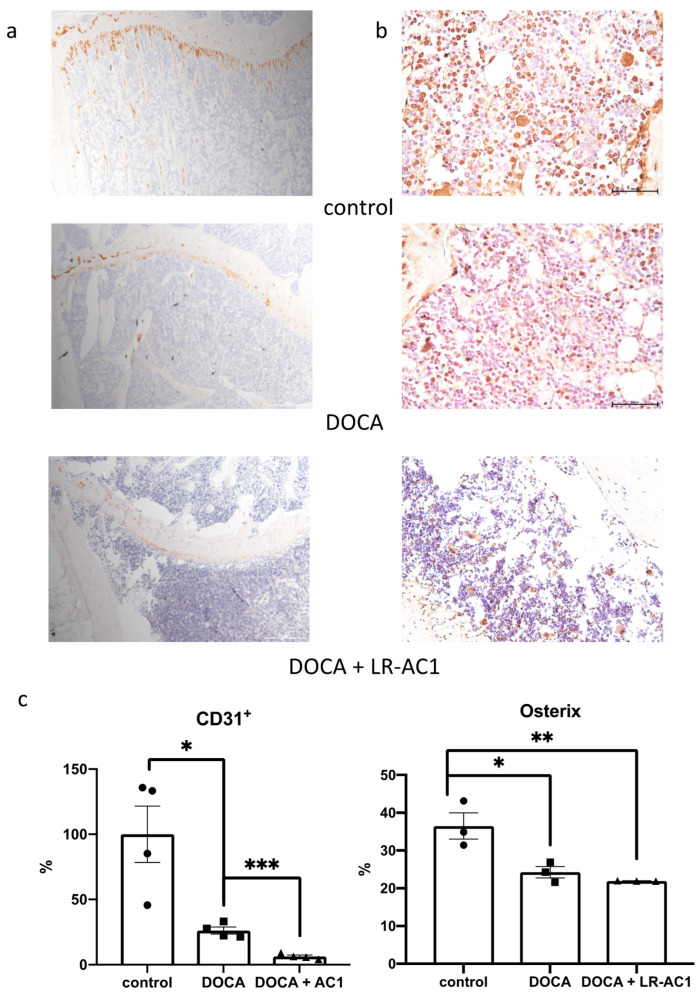
Effect of live LR-AC1 and DOCA on angiogenesis and osteogenesis in tibia primary spongiosa by immunostaining. Immunostaining of angiogenic biomarker CD^31+^ (**a**) and osteoclastogenic biomarker osterix (**b**) on the growth plate and primary spongiosa, respectively, of the tibia in the SHAM, DOCA rats, and DOCA rats supplemented with LR-AC1. Quantification of CD^31+^ and osterix expression in (**a**–**c**). The values are presented as mean ± SEM. Statistical analysis was conducted using one-way ANOVA, with significance levels indicated as follows: * 0.01 < *p* < 0.05; ** 0.001 < *p* < 0.01; *** *p* < 0.001.

To investigate the potential mechanism of bone loss following DOCA-salt-induced osteoporosis and LR-AC1 treatment, immunohistochemical (IHC) staining was performed targeting CD31^+^ and Osterix (Sp7). CD31^+^, also known as platelet endothelial cell adhesion molecule (PECAM-1), serves as an angiogenetic biomarker, while Osterix is a transcription factor for osteoblast differentiation [16,17]. Following DOCA induction, the immunolocalization of CD31^+^ was 26.16% of that observed in the control group, which is a significant decrease (*p* = 0.0145). LR-AC1 treatment resulted in an additional reduction, with the immunolocalization level of CD31^+^ dropping to only 6.36% of that in the control group (*p* = 0.0005) (Figure 3a). DOCA induction also led to a significant decrease in the immunolocalization of Sp7 (*p* = 0.0177), and LR-AC1 treatment could not ameliorate this effect (Figure 3b).

### 3.3. LR-AC1 CCS Hinders Osteoclastogenesis and Angiogenesis

**Figure 4:**
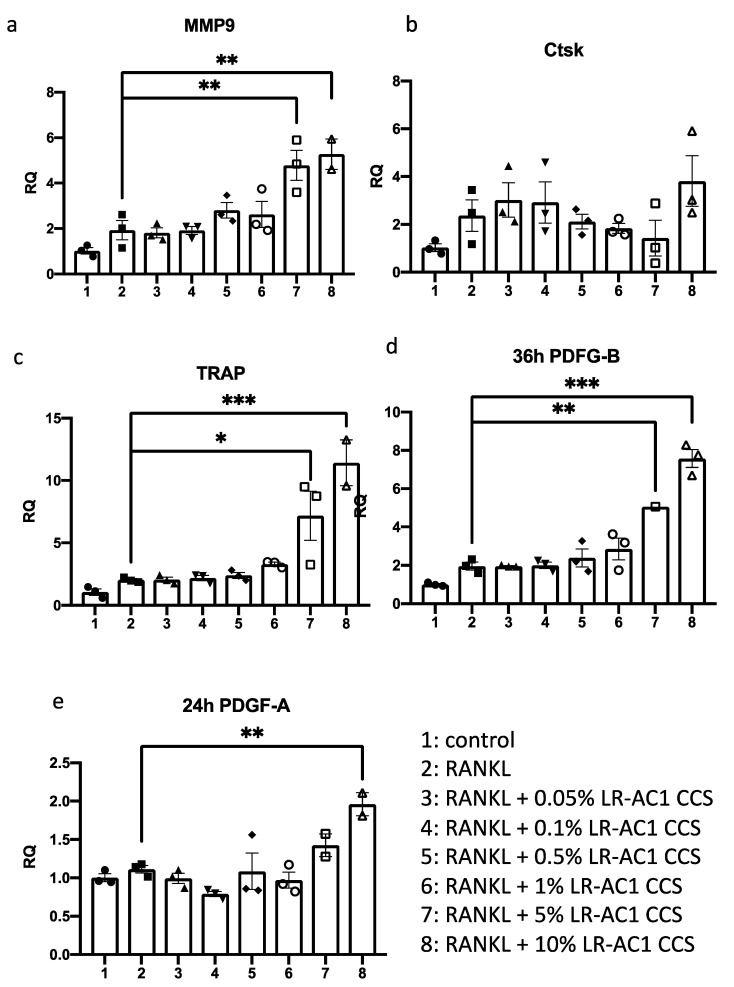
Effect of LR-AC1 CCS on osteoclastogenic and angiogenic biomarkers of RANKL-induced osteoclasts from RAW264.7. RT-qPCR was used to evaluate the expression of the osteoclastogenic biomarkers MMP9 (**a**), Ctsk (**b**), and TRAP (**c**), as well as the angiogenic biomarkers PDGF-A (**d**) and PDGF-B (**e**) in RANKL-induced RAW264.7 cells. MMP9, matrix metallopeptidase 9; Ctsk, Cathepsin K; TRAP, Tartrate-resistant acid phosphatase; PDGF-A, platelet-derived growth factor A subunit; PDGF-B, platelet-derived growth factor B subunit; RQ, relative quantity. The values are presented as mean ± SEM. Statistical analysis was conducted using one-way ANOVA, with significance levels indicated as follows: * 0.01 < *p* < 0.05; ** 0.001 < *p* < 0.01; *** *p* < 0.001.

RAW264.7 cells were induced to differentiate into osteoclasts by RANKL and subsequently co-incubated with varying concentrations of LR-AC1 CCS for 24 or 36 h. The cells were then collected for RNA extraction and RT-qPCR to determine the expression levels of osteoclast biomarkers, including MMP9, Ctsk, and TRAP. The transcript levels of all three biomarkers were nearly two-fold higher than the values before the RANKL induction, demonstrating successful differentiation of osteoclasts from the RANKL-treated RAW264.7 cells (Figure 4a–c). Treatment of the differentiated osteoclasts with LR-AC1 CCS resulted in dose-dependent increases in MMP9 and TRAP expression, suggesting that LR-AC1 CCS promoted osteoclastogenesis. A similar experimental setup was used for the RANKL-induced RAW264.7 cells treated with different concentrations of LR-AC1 CCS for 24 or 36 h to examine the effects on the angiogenesis markers PDGF-A and PDGF-B, respectively (Figure 4d,e). After 24 h of co-incubation, LR-AC1 CCS increased the expression of PDGF-A by 1.4 to 2.0-fold when the treatment concentration exceeded 5%. Similarly, the expression of PDGF-B nearly doubled following RANKL induction with 36 h of incubation. LR-AC1 CCS treatment at final concentration of 5% to 10% significantly increased the expression of PDGF-B in a dose-dependent manner. These results indicate that LR-AC1 CCS promoted angiogenesis during osteoclast differentiation.

### 3.4. The Interaction Between the LR-AC1 CCS-Treated RAW264.7 Cells and DOCA-Treated BMSCs Suppressed the Migration of BMSCs

**Figure 5:**
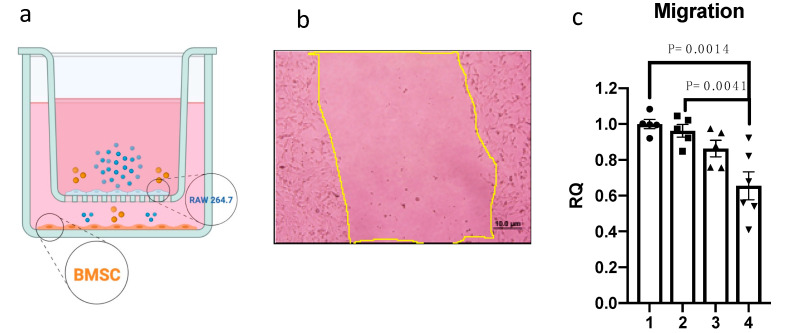
Effect of LR-AC1 CCS on the migration of BMSCs by wound healing assay. Design of RAW264.7 and BMSC co-culture (**a**), determination of migration by creating a wound and measuring the area after 24 h of healing time under a microscope (10×) (**b**), and the quantification results of the effect of DOCA and LR CCS on the migration of BMSCs (**c**). Statistical analysis was conducted using one-way ANOVA, and the values are presented as mean ± SEM. The cells were treated as shown in Table 2. BMSC, Bone mesenchymal stem cell; DOCA, Deoxycorticosterone acetate; LR-AC1 CCS, LR-AC1 cell-free conditioned supernatant.

A wound healing assay was conducted in a RAW264.7 and BMSC co-culture to investigate the effects of DOCA and LR-AC1 CCS on the migration of BMSCs. When 0.5 µmol/L of DOCA alone was applied to BMSCs, or when only 10% LR-AC1 CCS was used to treat RAW264.7 cells, no effect on BMSC migration was observed. In contrast, BMCS migration was significantly suppressed when RAW264.7 cells were treated with LR-AC1 CCS concurrent with BMSC treatment with DOCA, indicating that BMSC migration was inhibited by the interaction of the LR-AC1 CCS and DOCA treatments on RAW264.7 cells and BMSCs, respectively (Figure 5c).

### 3.5. Detrimental Effect of LR-AC1 Treatment on DOCA-Induced Bone Loss Associated with Altered Gut Microbiota

**Figure 6:**
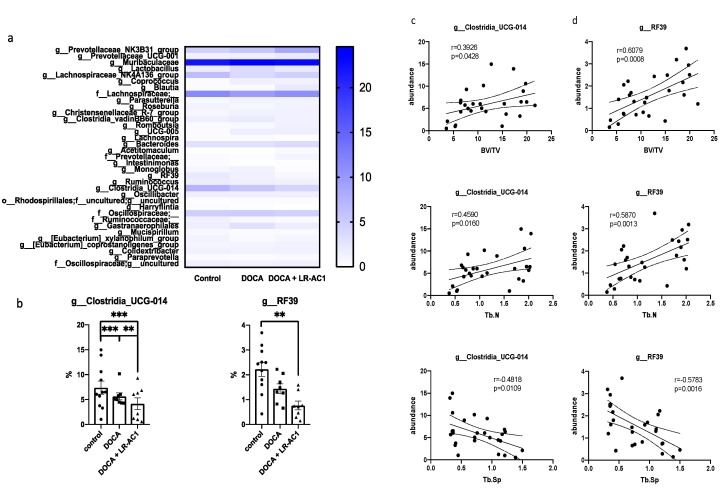
Gut microbiota of 16S rRNA sequencing. Significant changes in relative abundance between control group, DOCA induction group, and LR-AC1 treatment group at genus level (**a**) and selected genera showing same trend as bone loss in this project (**b**). Abundances of two selected genera were significantly positively correlated with BV/TV and Tb.N and were negatively related to Tb.Sp (**c**,**d**). The values are presented as mean ± SEM. Statistical analysis was performed using one-way ANOVA, with significance levels indicated as follows: ** 0.001 < *p* < 0.01; *** *p* < 0.001.

Analysis of 16S rRNA sequencing revealed significant differences in the relative abundances of 35 bacteria genera among the three groups (Figure 6a,b). The relative abundances of genera *g_RF39* and *g_Clostridia_UCG-014* exhibited trends consistent with the bone loss observed in the control, DOCA treatment, and LR-AC1 treatment groups. Interestingly, the relative abundances of these two genera showed positive correlations with the BV/TV and Tb.N of the primary spongiosa (Figure 6c,d). Conversely, negative corrections between the relative abundances of these genera and the Tb.Sp of the primary spongiosa were observed (Figure 6c,d). These findings suggest that the gut microbiome may influence bone metabolism. Specifically, *g_RF39 and g_Clostridia_UCG-014* may play important roles in maintaining bone structure, and reductions in these two genera are associated with the bone loss phenotype. Given the limited information in this study, further investigation is needed to determine whether changes in these two bacterial genera have a causal relationship with bone loss.

## 4. Discussion

In this study, DOCA-salt treatment resulted in bone loss characterized by a reduction in trabecular bone, a decrease in osteoblasts, and impaired angiogenesis. LR-AC1 CCS increased the expression of osteoclastogenic and angiogenic markers, suggesting that LR-AC1 can enhance bone resorption and induce angiogenesis in vitro. The interaction between LR-AC1 CCS treatment on RAW264.7 cells and DOCA treatment on BMSCs inhibited BMSC migration. In vivo, live LR-AC1 aggravated bone loss in rats with DOCA salt induced osteoporosis by reducing osteogenesis and inhibiting angiogenesis but had no effect on healthy rats. Changes in gut microbiota were associated with DOCA-salt-induced bone loss, with the relative abundances of the genera *g_RF39* and *g_Clostridia_UCG-014* showing trends consistent with bone loss and correlating with the bone parameters.

In this study, we demonstrated that DOCA salt induced both osteoporosis and hypertension. DOCA, a glucocorticoid, is associated with significant adverse effects, including bone loss. The possible mechanisms through which glucocorticoids induce osteoporosis are attributed to promoting osteoblast and osteocyte apoptosis, altering the gut microbiota composition, and undermining the intestinal barrier [18]. Glucocorticoids reduce bone mass by affecting bone formation and bone resorption and calcium deficiency. Furthermore, glucocorticoids exert stress on the endocrine system, leading to irregular hormone regulation, including decreased osteoblast synthesis, reduced androgen secretion, and diminished growth hormone levels, all of which inhibit bone formation [19]. Our findings indicate that DOCA salt treatment reduced the expression of osterix, impacting osteoblast differentiation and consequently inhibiting bone formation. Additionally, glucocorticoids increase parathyroid hormone secretion and enhance osteoclast activity, further promoting bone resorption [19]. We performed TRAP staining to investigate the effects of DOCA salt treatment on osteoclasts; however, the results were inconclusive. Calcium absorption in the gut could be affected by glucocorticoid treatment, which leads to calcium deficiency. Since calcium absorption in the gut was not measured in this study, the impact of DOCA on calcium absorption remains unknown. Based on previously published findings, steroid-induced osteoporosis could potentially be treated by modulating bone metabolism and supplementing calcium [19].

Supplementation with probiotics, including *Lactobacillus* spp., is considered a preventive strategy or even therapeutic option for osteoporosis. The *Lactobacillus* spp. used in this study was a strain of *L. rhamnosus*. Previous studies demonstrated that certain strains of *L. rhamnosus* can attenuate osteoporosis. For example, Leena Sapra et al. showed that treatment with *L. rhamnosus* enhanced the expression of anti-osteoclastogenic cytokines while reducing the expression of osteoclastogenic cytokines in an ovariectomy (OVX)-induced mouse model of osteoporosis. Additionally, in a postmenopausal mouse model, bone loss was mitigated by *L. rhamnosus,* as observed following various analyses. The inhibition of osteoclastogenesis by *L. rhamnosus* also influenced the differentiation of Treg-Th17 cells. Furthermore, *L. rhamnosus* treatment reduced the percentage of osteoclastogenic CD4^+^Rorγt^+^Th17 cells and increased the percentage of anti-osteoclastogenic CD4^+^Foxp3^+^Tregs and CD8^+^Foxp3^+^Tregs at distinct immunological sites [20]. Jau-Yi et al. utilized gonadotropin-releasing hormone (GnRH) agonists, specifically Lupron Depot, to induce a sex-steroid-deficiency osteoporosis model in both conventional and germ-free mice. The researchers reported that this induction led to increased gut permeability and promoted the expression of osteoclastogenic cytokines. Gut microbiota appeared to be an important factor driving bone loss in their study. Interestingly, another *L. rhamnosus* strain GG (LGG) was shown to enhance gut barrier integrity, thereby protecting bone from sex steroid deficiency [21]. Zhou’s group further observed that LGG had a positive effect on tenofovir disoproxil fumarate (TDF)-induced bone loss, demonstrating that LGG could modulate the gut microbiota, improve intestinal integrity, and inhibit the inflammatory response associated with TDF treatment [22]. Overall, *L. rhamnosus* has been shown to exert beneficial effects on various types of osteoporosis.

Similarly to previous studies involving other *L. rhamnosus* strains, this study utilized *L. rhamnosus* strain AC1, isolated in Hong Kong, for the treatment of osteoporosis, followed by micro-CT analysis and H&E staining to observe the bone phenotype. In contrast to the work of Leena Sapra et al., which focused on both cortical and trabecular bone in lumbar vertebrae-5, the tibia, and the femur, our study concentrated solely on the trabecular bone of the tibia [20]. Unexpectedly, LR-AC1 not only failed to improve bone loss induced by DOCA but also aggravated bone loss. Unlike the positive effects reported in previous studies using other *L. rhamnosus* species in OVX-, sex-steroid-deficiency-, and TDF-induced osteoporosis models, LR-AC1 appeared to negatively impact DOCA-induced osteoporosis [20,21,22]. Various studies have demonstrated that probiotic supplementation has positive effects on bone loss induced by OVX surgery. However, some findings suggest that these effects may be strain-specific, for instance, treatment with *L. plantarum AR237*, *L. paracasei*, or a mixture of *Lactobacillus* species showed no effect on femur bone mineral density (BMD) [23,24]. We conducted staining of bone tissues and in cellulo experiments to investigate osteoclastogenesis and osteoblastogenesis. Both sets of results suggested that LR-AC1 treatment promoted osteoclastogenesis, although its effect on osteoblastogenesis remains undetermined. As angiogenesis plays a critical role in bone regeneration, we also examined the status of osteoblastogenesis and angiogenesis instead of osteoclastogenesis in our samples. Coupling of angiogenesis and osteogenesis contributes to bone development and bone repair [25]. Type H vessels, which are in proximity to the growth plate, exhibit high levels of expression of CD31^+^ and regulate the proliferation and differentiation of osteoblasts and osteoclasts. Our study revealed a reduction in CD31^+^ expression following osteoporosis induction with DOCA, which was further diminished by live LR-AC1 treatment. Intriguingly, when RAW264.7 cells were induced to differentiate into osteoclast, the LR-AC1 CCS treatment increased the expression of PDGF, indicating that LR-AC1 CCS may promote angiogenesis. PDGF-BB, which is secreted by preosteoclasts, contributes to the migration and differentiation of mesenchymal stem cells and endothelial progenitor cells, promoting angiogenesis and osteogenesis. It has been established that glucocorticoids inhibit PDGF-BB secretion, which suppresses angiogenesis and reduces osteogenesis [26]. The results of our wound healing assay indicated that the interaction between DOCA and LR-AC1 CCS suppressed the migration of BMSCs in a co-culture with RAW264.7 cells. Live LR-AC1 treatment in rats with DOCA salt-induced osteoporosis might impair the migration of BMSCs which, in turn, suppresses angiogenesis and eventually aggravates bone loss.

We also explored the effect of LR-AC1 treatment on the alteration of gut microbiota. In this study, *Clostridia-UCG-014* exhibited a significant positive association with the phenotype of bone loss. A previous study demonstrated that *Clostridia-UCG-014* is an important genus associated with tryptophan metabolism [27]. Two pathways of tryptophan metabolism, namely the kynurenine pathway and serotonin pathway, have been reported to be associated with bone biology [28]. The kynurenine pathway has complex effects on osteoblastogenesis. For instance, 3-hydroxykynurenine (3-HKYN), a known metabolite from the kynurenine pathway, reduces the activity of osteoblasts. On the other hand, the end products of this pathway, such as 3-hydroxyAA, xanthurenic acid, picolinic acid, quinolinic acid, and NAD^+^, play positive roles in bone formation. Serotonin also has varying impacts on osteogenesis [29,30]. Gut-derived serotonin inhibits bone formation by reducing osteoblastogenesis and increasing bone resorption. However, brain-derived serotonin enhances bone formation and inhibits bone resorption, positively affecting bone mass [31]. In our results, the relative abundance of *Clostridia-UCG-014* followed a trend consistent with bone loss. Therefore, treatment with live LR-AC1 in DOCA-salt-induced osteoporosis altered the composition of the gut microbiota and reduced the relative abundance of *Clostridia-UCG-014*, which may, in turn, have affected tryptophan metabolism and disturbed bone homeostasis.

A culture supernatant of LGG was utilized to improve calcium deficiency by promoting the intestinal absorption of vitamin D in senile osteoporosis. The LGG supernatant upregulated vitamin D transporters, enhancing the absorption of cholecalciferol in the intestine and increasing the levels of 25OHD3 in vivo and in vitro [32]. Although vitamin D absorption was not measured in this study, the results indicated that LR-AC1 treatment aggravated bone loss, suggesting LR-AC1 has limited or no effect on bone loss, regardless of calcium deficiency. Overall, these findings suggest that bone formation and resorption may be the critical factors in osteoporosis in our study.

Several considerations and limitations of this study should be acknowledged. First, this study was conducted exclusively in male rats using a DOCA-salt-induced osteoporosis model, and the observation period was limited to the duration of the intervention without extended follow-up assessment. The use of CCS may not perfectly reproduce the in-vivo metabolites of LR-AC1 in the rats. Additionally, the bone analysis focused specifically on the trabecular bone tissue of the tibia, and cortical bone parameters and other skeletal sites, including the spine and femur, were not evaluated in this investigation. Furthermore, the potential relationships between alive LR-AC1 administration and gut health parameters, particularly gut barrier integrity and its possible influence on bone metabolism, require additional investigation to better understand the mechanistic pathways involved. Last but not least, although this study identified associations between live LR-AC1 treatment and tryptophan metabolism, the detailed mechanisms underlying these metabolic changes and their subsequent effects on bone homeostasis remain to be fully elucidated in future research.

## 5. Conclusions

In this study of a male rat model of DOCA-salt-induced osteoporosis, live LR-AC1 suppressed the migration of BMSCs, which may have disrupted the coupling of angiogenesis and osteogenesis, thereby aggravating bone loss. Moreover, live LR-AC1 treatment reduced the abundance of *Clostridia-UCG-014*, which is associated with tryptophan metabolism, and may have contributed to bone loss. Various studies have reported that probiotics could serve as potential preventive and therapeutic agents for osteoporosis in various models and across different genders. However, live LR-AC1 did not effectively improve osteoporosis induced by DOCA salt in male rats, underscoring the need for scientific evidence to support the beneficial function of probiotics in various diseases when selecting probiotic strains as treatment options. Healthcare providers should recommend probiotic strains based on documented pre-clinical or clinical evidence of efficacy for specific disease indications rather than assuming universal benefits across all strains for all conditions.

## Figures and Tables

**Table 1:** Primer sequences.

Primer Name	Reverse	Forward
PDGF-A	TGTTCAGGAATGTCACACGCC	GCAAGACCAGGACGGTCATTTAC
PDGF-B	CTTCTTTCGCACAATCTCAAT	CACTCCATCCGCTCCTTT
MMP9	AGAGTACTGCTTGCCCAGGA	CGTCGTGATCCCCACTTACT
Ctsk	TTTCCTCCGGAGACAGAGCA	AGCACCCTTAGTCTTCCGCT
TRAP	GGTAGTAAGGGCTGGGGAAG	CTGCTGGGCCTACAAATCAT
GAPDH	GGCATGGACTGTGGTCATGA	CAACTCCCTCAAGATTGTCAGCAA

MMP9, matrix metallopeptidase 9; Ctsk, Cathepsin K; TRAP, Tartrate-resistant acid phosphatase; PDGF-A, platelet-derived growth factor A subunit; PDGF-B, platelet-derived growth factor B subunit; GAPDH, Glyceraldehyde-3-phosphate dehydrogenase.

**Table 2:** Cell treatment conditions.

	1	2	3	4
RAW264.7	10% FBS DMEM	10% FBS DMEM	10% LR-AC1 CCS	10% LR-AC1 CCS
BMSC	10% FBS 1% DMSO DMEM	0.5 µmol/L DOCA	10% FBS 1% DMSO DMEM	0.5 µmol/L DOCA

BMSC, bone mesenchymal stem cell; DOCA, deoxycorticosterone acetate; LR-AC1 CCS, LR-AC1 cell-free conditioned supernatant.

## Data Availability

The data that support the findings of this study are available from the corresponding author upon reasonable request.

## Abbreviations

The following abbreviations are used in this manuscript:

LR-AC1	*Lactobacillus rhamnosus* AC1
CCS	Cell-free conditioned supernatant
BMSCs	Bone marrow mesenchymal stem cells
MRS	De Man, Rogosa, and Sharpe
rRNA	Ribosomal RNA
SD	Sprague Dawley
CAFs	Centralized Animal Facilities
PFA	Paraformaldehyde
EDTA	Ethylene Diamine Tetraacetic Acid
DAB	Diaminobenzidine
DMEM	Dulbecco’s modified Eagle medium
PBS	Phosphate-buffered saline
FBS	Fetal bovine serum
RANKL	Nuclear factor kappa-B ligand
MMP-9	Matrix metallopeptidase
Ctsk	Cathepsin K
TRAP	Tartrate-resistant acid phosphatase
PDGF-A	Platelet-derived growth factor A
PDGF-B	Platelet-derived growth factor B
RT-qPCR	Reverse transcriptase quantitative polymerase chain reaction
BV/TV	Bone volume to total volume
Tb.Th	Trabecular thickness
Tb.N	Trabecular number
Tb.Sp	Trabecular separation
GnRH	Gonadotropin-releasing hormone
LGG	*L. rhamnosus* strain GG
TDF	Tenofovir disoproxil fumarate
3-HKYN	3-hydroxykynurenine
